# An Advanced Entropy Approach for Minimizing False Discoveries in Imputation-Based Association Analyses

**DOI:** 10.21203/rs.3.rs-8264218/v1

**Published:** 2025-12-17

**Authors:** Zhihui Zhang, Dakai Zhu, Xiangjun Xiao, Christopher I. Amos

**Affiliations:** 1 Dan L Duncan Comprehensive Cancer Center, Baylor College of Medicine, Houston, TX, USA; 2 Institute of Clinical and Translational Medicine, Department of Medicine, Baylor College of Medicine, Houston, TX, USA; 3 Department of Internal Medicine, Division of Epidemiology, Biostatistics, and Preventive Medicine, UNM Comprehensive Cancer Center, Albuquerque, NM, USA; 4 Department of Genetics, MD Anderson Cancer Center, Houston, TX, USA

**Keywords:** Genotype imputation, GWAS power and FDR, Entropy weighting, Dosage-based methods

## Abstract

Genotype imputation is a cornerstone of modern genetic studies, enhancing the resolution of genome-wide association studies (GWAS), fine mapping, and polygenic risk score estimation by inferring untyped variants using reference panels. The output of imputation is a set of probabilistic genotypes, each associated with an inherent degree of uncertainty. However, conventional downstream analyses often overlook this uncertainty, relying instead on allelic dosages—expected allele counts computed from probabilistic genotypes—as proxies. This practice can be misleading, as distinct genotype probability distributions may produce identical dosages despite vastly different confidence levels, potentially introducing bias and inflating false discoveries. To address this limitation, we introduce an entropy-weighted association method that explicitly quantifies imputation uncertainty using Shannon entropy. These entropy values are integrated as observation-level weights within the association model, allowing the method to dynamically account for the reliability of each imputed genotype. Through simulation studies, we demonstrate that this approach substantially reduces false positives, especially when genotypic uncertainty is pronounced. Our findings highlight the importance of modeling imputation uncertainty and offer a framework that improves the robustness of GWAS and other genotype imputation-dependent analyses.

## Background

Missing genotype data is a prevalent challenge in genetic studies, particularly genome-wide association studies (GWAS), arising when certain single nucleotide polymorphisms (SNPs) are not captured by genotyping platforms. This issue is especially pronounced in studies using sparse marker arrays, combining datasets from multiple platforms, or employing cost-effective sequencing methods such as low-coverage whole-genome sequencing (lcWGS).^1,2^

Genotype imputation, powered by high-density public reference panels and advances in next-generation sequencing, has become essential for overcoming these limitations. It enables the inference of untyped variants in sparse datasets, substantially enhancing statistical power, enabling integration across heterogeneous studies, and maximizing the value of existing genetic resources.^3,4^ Successful applications of genotype imputation have led to numerous discoveries in GWAS, including novel disease loci^5–10^, and have facilitated fine-mapping^9,11^, causal rare variant discovery^12–14^, and polygenic risk score estimation^12–14^. Despite these advances, imputed genotypes are inherently probabilistic, carrying uncertainty influenced by factors such as the array density, reference panel quality, sequencing depth, population structure, and allele frequency. If not properly accounted for, this uncertainty can introduce biases and increase false-positive rates, undermining the reliability of genetic associations.^15–19^

Current practices in GWAS typically manage imputation output and uncertainty by converting probabilistic genotypes into expected allele counts, or “allelic dosages,” and analyzing these dosages using standard regression models. However, the same dosage value can result from genotype probability distributions with markedly different uncertainty levels. Consequently, treating dosage values as direct proxies for true genotypes can misrepresent uncertainty, leading to biased association results. Indeed, our previous evaluation using UK Biobank data revealed widespread p-value inflation—particularly in datasets derived from sparse genoytping—accompanied by an elevated false discovery rate. These artifacts were associated with factors such as poor imputation quality, low minor allele frequency (MAF), high genotype missingness, and imbalanced case-control ratios.^19^

To address these critical limitations, we introduce a novel entropy-based association method grounded in information theory. This approach explicitly quantifies imputation uncertainty at the genotype level using Shannon entropy^20^ and incorporates these values as observation-specific weights within the association model. To rigorously evaluate the performance of our entropy-weighted method in comparison to the traditional dosage-based approach, we conducted extensive simulations using both dense genotype data from the 1000 Genomes Project^21^ and sparse array-based data from the UK Biobank^22^. By applying a range of masking ratios, we generated datasets spanning a wide spectrum of SNP densities—from high-resolution WGS-like data to sparse configurations comparable to consumer genotyping arrays.

Across these diverse scenarios, we evaluated performance metrics including statistical power, false discovery rates, imputation quality scores (R^2^), and minor allele frequency (MAF) stratification. In addition, we analyzed p-value drift, measuring the extent to which imputation uncertainty distorts association signals relative to those derived from complete, unmasked data. Our results demonstrate that the entropy-weighted method consistently outperforms the dosage-based approach—not only by reducing false positives and improving signal stability, but also by minimizing the divergence of imputed p-values from their complete-data counterparts. This advantage becomes particularly pronounced under extreme sparsity, where traditional methods are most vulnerable to uncertainty-induced inflation. Together, these findings highlight the robustness and adaptability of the entropy-weighted framework for imputation-based genetic analyses, especially in real-world settings where genotype density and data quality vary widely.

## Methods

### Uncertainty Quantification

For each imputed diallelic marker, standard imputation software estimates probabilistic genotypes corresponding to the three possible genotype categories: homozygous common (AA), heterozygous (Aa), and homozygous rare (aa). These probabilistic genotypes are expressed as posterior probabilities ${(P}_{AA},P_{Aa},P_{aa})$, The widely used dosage method summarizes these probabilistic genotypes into a single numeric value known as the allelic dosage, calculated as $P_{Aa}+2P_{aa}$. However, distinct probabilistic genotypes with differing certainty levels can yield identical dosage values. For example, the genotype sets (0.33, 0.33, 0.33) and (0, 1, 0) both have a dosage of 1, despite the second set being clearly more certain. To quantify this overlooked uncertainty, we adopted the concept of entropy from information theory, specifically Shannon entropy^20^. Shannon entropy H(X) for a discrete random variable X with possible outcomes $x_{1},\ldots ,x_{n}$, occurring with probabilities $P\left(x_{1}\right),\ldots ,P\left(x_{n}\right)$, is defined as:

$$H\left(X\right)=-\sum_{i=1}^{n}P\left(x_{i}\right)\log_{b}P\left(x_{i}\right),$$

In the context of genotype imputation, the outcomes $x_{i}$ represent the three possible genotypes and $P\left(x_{i}\right)$ their respective probabilities. Entropy values range from approximately −1.58 (maximum uncertainty) to 0 (complete certainty). To incorporate this uncertainty into the association analysis, we applied the transformation $w_{i}=1-\frac{H_{i}}{{log}_{2}(3)}$ and rescaled the entropy values to a [0, 1] range. This mapping ensures that fully uncertain genotypes (maximum entropy) receive a weight close to 0, while highly certain genotypes (low entropy) are assigned weights near 1.

### Statistical Association Model

Association analyses were conducted using logistic regression models for binary traits. The likelihood for individual i incorporating the entropy-based weight $\omega_{i}$ was defined as:

$$l=\sum_{i=1}^{n}w_{i}\left[y_{i}\text{logP}\left(y_{i}=1|x_{i},G_{i}\right)+\left(1-y_{i}\right)\text{log}\left(1-\text{logP}\left(y_{i}=1|x_{i},G_{i}\right)\right)\right]$$

where $y_{i}$ represents the binary phenotype, $x_{i}$ is the vector of covariates, and $G_{i}$ is the genotype dosage for individual i. Parameters were estimated using maximum likelihood, and statistical significance was assessed via likelihood ratio tests (LRT). Model fitting and testing were implemented using functions from the R package lmtest^23^.

### Simulation Design and Data

#### Genotype Data Simulation

To evaluate the performance of our entropy-weighted association method, we designed simulation studies using real genotype data from the 1000 Genomes Project (1000G)^21^. Specifically, we focused on chromosome 1, which includes approximately 6.2 million SNPs across 2,504 individuals. Quality control (QC) filtering was applied to exclude SNPs with Hardy-Weinberg equilibrium (HWE) p-values below 1 × 10^−5^ and those with missingness rates exceeding 1%, yielding a high-quality dataset for simulation.

From this dataset, we randomly selected 1,000 unrelated individuals as our study cohort, balanced equally between 500 cases and 500 controls. To simulate phenotypes, we first applied linkage disequilibrium (LD) pruning using PLINK2^24^ to retain a set of independent SNPs. To simulate phenotypes, we first selected 100 causal SNPs from an LD-pruned subset of variants. For each causal SNP, an effect size $\left(\beta_{g}\right)$ was assigned by sampling from a normal distribution $\beta_{g}\sim N\left(0.3,0.1^{2}\right)$ to ensure causal variants had moderately strong effect sizes to enable clear signal detection in association analyses. Non-causal SNPs were assigned an effect size of zero. Using these effect sizes, we simulated binary phenotypes under a logistic model:

$$\text{logit}\left(P\left(y_{i}=1|G_{i}\right)\right)=\mu +\sum_{j=1}^{100}\beta_{gi}G_{ij}$$

where $G_{i}$ denotes the genotype value (allelic dosage) for SNP j in individual i, μ is an intercept term. The linear predictor $\left(\eta_{i}\right)$ was converted to a probability, $p_{i}$ using the inverse logit function:

$$p_{i}=\frac{e^{\eta_{i}}}{1+e^{\eta_{i}}}$$

where $\eta_{i}=\mu +\sum_{j=1}^{100}\beta_{gi}G_{ij}$. To ensure a balanced design with equal numbers of cases and controls, we randomly selected 500 cases and 500 controls for the final study cohort. To mimic real-world data sparsity encountered in genotyping arrays and low-pass sequencing, we systematically masked genotype data at rates from 30% to 90%, resulting in varying SNP densities from ~17,400 to ~2,500 SNPs/Mb.

#### Extreme Sparse Genotype Data Simulation

To further benchmark our method under extreme data sparsity, we conducted additional simulations using genotype array data from the UK Biobank^22^. The UK Biobank genotyping array includes approximately 805,000 directly genotyped variants, corresponding to an average density of about 280 SNPs per megabase across the genome. Chromosome 1 genotypes were extracted and filtered using the same QC criteria (HWE p < 1 × 10^−5^, SNP and sample missingness <5%), resulting in a dataset of 42,691 high-quality SNPs. Related individuals were removed based on kinship analysis using KING^30^, retaining only unrelated participants. We randomly selected 20,000 unrelated individuals as the study cohort, maintaining a balanced 1:1 case-control ratio. An additional 80,000 unrelated individuals were used as the reference panel for imputation. We then randomly masked 20%, 40%, 60%, and 80% of SNPs—corresponding to approximate SNP densities of 200, 150, 100, and 50 SNPs/Mb, respectively.

Using PLINK2, we first performed association testing on the complete dataset to obtain “truth set” p-values for each SNP (P_nomiss_). To confirm that these p-values were well-calibrated and not inflated under the null, we examined their distribution using a QQ plot (Supplement Figure 1), which showed no deviation from expectation, supporting the validity of this baseline. Genotypes were phased and imputed using Beagle v5.4^25^, chosen for its robust accuracy and efficient runtime compared to tools such as Minimac or IMPUTE2^25^. The imputation yielded both allelic dosage values and probabilistic genotype distributions, which were subsequently used for association testing via the dosage-based and entropy-weighted models.

#### Performance Evaluation

**Figure 1:**
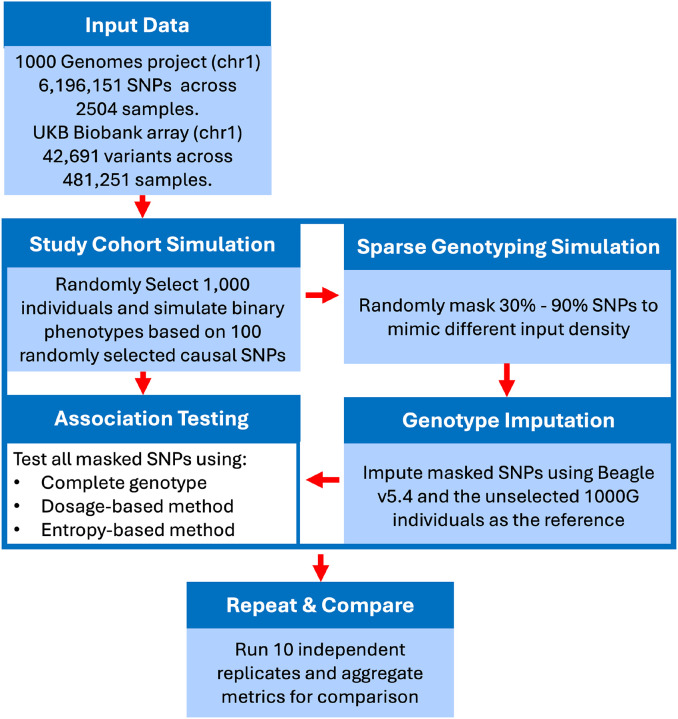
Illustrative Workflow Diagram of Conducted Simulations in the Study

To evaluate the effectiveness of different association methods, we compared three sets of analyses across multiple simulation replicates: (1) the complete genotype dataset (unmasked “true” data), (2) imputed genotype dosages, and (3) our proposed entropy-weighted imputation method. For each approach, we performed logistic regression analyses to obtain p-values. Performance was assessed by examining the discordance between the imputed methods’ p-values (dosage and entropy-weighted) and the true genotype-based p-values, calculated as −log10(P_dosage_/P_nomiss_) and −log10(P_weight_/P_nomiss_), respectively. To further validate imputation quality, we utilized the allelic R^2^ metric provided by Beagle v5.4^25^, which quantifies the correlation between true and imputed genotypes, with values ranging from 0 (poor) to 1 (perfect imputation quality). The entire simulation workflow—including the selection of individuals, masking, imputation, and association testing—was independently repeated 10 times to ensure robustness. Final conclusions and comparisons were drawn from the aggregated results across all replicates. Figure 1 provides a comprehensive illustration of our simulation and analytical pipeline.

## Results

**Figure 2:**
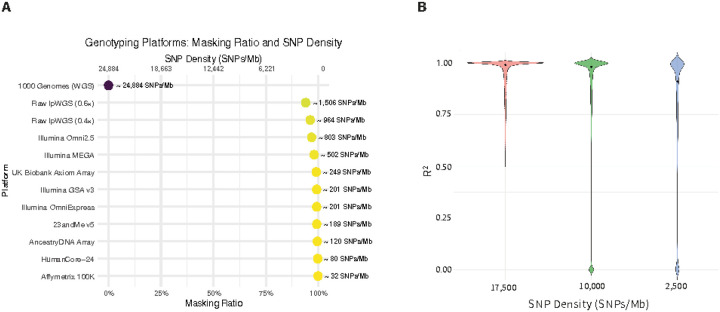
Comparison of Simulated Masking Ratios and Imputation Performance Across Genotyping Densities (A) This panel compares the simulated masking ratios used in our study with the SNP densities of widely used genotyping and sequencing platforms, based on chromosome 1. Each point represents a platform, annotated with its masking ratio (relative to the 1000 Genomes reference) and corresponding SNP density in SNPs per megabase. The highest density corresponds to the unmasked 1000G reference (~24,884 SNPs/Mb), while sparse consumer genotyping arrays such as 23andMe (~189 SNPs/Mb) and AncestryDNA (~120 SNPs/Mb) approximate masking levels above 99%. Mid-range platforms like the UK Biobank Axiom array (~249 SNPs/Mb) align with ~90% masking, and low-pass WGS (0.4×–0.6×) ranges between ~964 and ~1,506 SNPs/Mb. (B) This panel illustrates the relationship between genotype sparsity and imputation quality, quantified by the distribution of allelic R^2^ values. SNPs with high imputation quality (R^2^>0.5) in a dense input dataset (SNP density: 17,500 SNPs/Mb) were identified and their corresponding R^2^ values were evaluated in a sparser dataset with substantially lower input density. The comparison reveals a downward shift in imputation quality under sparse conditions, with many previously high-quality SNPs exhibiting reduced R^2^ values, including a notable proportion with R^2^=0.

To contextualize our simulation framework, we compared the SNP densities resulting from our masking strategy with those of widely used genotyping platforms (Figure 2A). The SNP density of the UK Biobank Axiom array, for example, is approximately 249 SNPs/Mb on chromosome 1—representing just about 1% of the variant density observed in the 1000 Genomes Project (WGS), which has nearly 25,000 SNPs/Mb. Consumer-facing arrays such as 23andMe (~189 SNPs/Mb) and AncestryDNA (~120 SNPs/Mb) offer even sparser coverage. While low-pass whole-genome sequencing (lpWGS, 0.4×–0.6×) provides higher densities (~1,000–1,500 SNPs/Mb), it still captures only a small fraction of the information available in high-resolution reference panels. These density estimates are approximate and may vary across versions and coverage. In practice, the number of usable SNPs is often further reduced after applying quality control filters that remove variants with high missingness or poor quality. Our masking strategy, which generated datasets with SNP densities ranging from approximately 17,500 to 50 SNPs/Mb, closely aligns with the input sparsity observed in these real-world datasets, demonstrating that our simulation design realistically reflects the range of genotyping densities encountered in practice.

We then evaluated how input SNP density influences imputation accuracy using the allelic R^2^ metric (Figure 2B). As masking ratios increased, imputation quality declined sharply. In particular, we tracked how SNPs that were well imputed in dense datasets (R^2^>0.5) progressively lost quality as input density decreased. Strikingly, many of these same SNPs exhibited a complete collapse in confidence, with their R^2^ values dropping to zero under ultra-sparse conditions. Importantly, as shown in Figure 2A, several widely used genotyping platforms—including consumer-grade arrays—have even lower SNP densities than the simulated datasets used in Figure 2B. This suggests that, in practice, imputation quality from such platforms may degrade even further than what we observed in Figure 2B. It is worth noting that in our simulation design, the study samples and reference samples were drawn from the same cohort and had a perfectly balanced case-control ratio, conditions that are favorable for imputation accuracy. In real-world studies, however, reference panels and study cohorts often differ in ancestry composition, sequencing quality, or case-control imbalance, factors that can substantially lower imputation performance. Therefore, our simulation results likely represent an optimistic baseline; the impact of genotype uncertainty is likely to be even more pronounced in real-world studies than in our controlled simulations.

**Figure 3:**
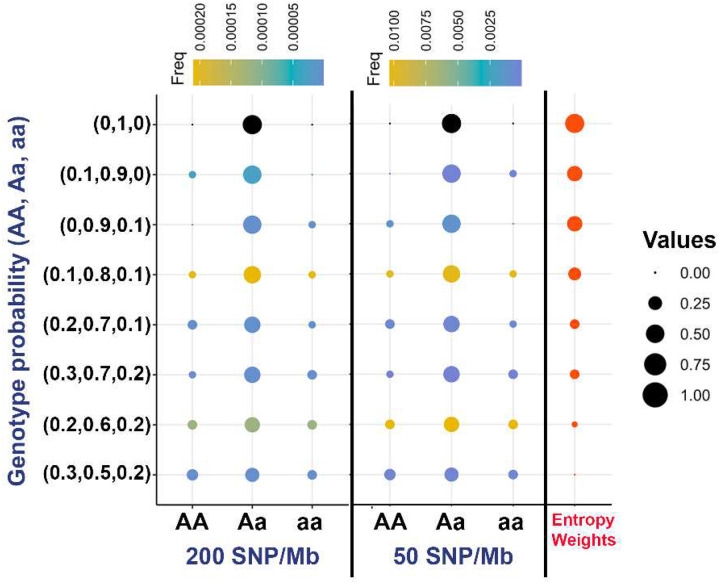
Distribution of diverse posterior probability sets of genotypes. This figure illustrates how genotype posterior probability distributions differ between datasets generated at two input SNP densities: ~200 SNPs/Mb (left) and ~50 SNPs/Mb (right). We focus on SNPs with dosages between 0.9 and 1.1. For each SNP, genotype posterior probabilities Pr(AA, Aa, aa) were grouped into discrete probability patterns and aggregated across all individuals from 10 independent simulation replicates. From top to bottom, the probability patterns are ordered by decreasing heterozygous posterior probability Pr(Aa). Circle color indicates the frequency of each probability pattern, while circle size reflects the magnitude of the posterior probabilities and their associated entropy-based weights (red circles in the rightmost column; values rounded to one decimal place). The fully confident heterozygous pattern (0, 1, 0) — shown at the top of the left and middle panels — is excluded from the frequency summaries to highlight the diversity of less-certain genotype calls.

Figure 3 presents 8 representative genotype probability sets (Pr(AA), Pr(Aa), Pr(aa)) identified from simulation studies under two levels of input SNP density: approximately 200 SNPs/Mb and 50 SNPs/Mb, respectively. Each row corresponds to a distinct probabilistic cluster, derived by rounding posterior genotype probabilities to one decimal place to streamline classification. Without rounding, a far more complex spectrum of genotype probability profiles would be observed. The left and center panels illustrate the distribution of posterior genotype probabilities across these groups for datasets in different SNP densities. From the top to the bottom of the figure, the probability of the heterozygous genotype (Pr(Aa)) declines, reflecting increasing uncertainty in the imputed genotypes. For example, the bottom group, which has posterior probabilities (Pr(AA), Pr(Aa), Pr(aa)) = (0.3, 0.5, 0.2), becomes far more prevalent as input SNP density decreases—from a frequency of 2.1 × 10^−6^ at 200 SNPs/Mb to 2.9 × 10^−4^ at 50 SNPs/Mb, representing a >100-fold increase. This proliferation of ambiguous genotypes with lower SNP density leads to greater noise in downstream association tests, which contributes to elevated false discovery rates. Notably, the dosage values for all 8 clusters fall within a narrow band (~0.9 to 1.1), limiting their capacity to differentiate levels of uncertainty. In contrast, the entropy-derived weights, shown in the right panel, offer a more interpretable and sensitive metric of uncertainty. As the genotype distributions become more ambiguous, the corresponding weights drop from 1 (high certainty) to 0 (high uncertainty), effectively stratifying probabilistic genotypes that appear indistinguishable using dosage alone.

**Figure 4:**
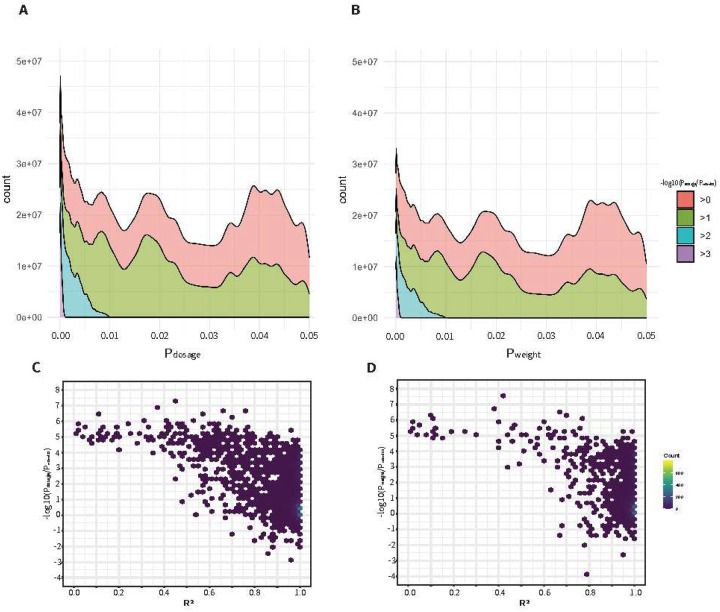
Comparison of p-value Discordance Panels A and B display the relationship between imputed p-values (x-axis: P_method_) and the discordance between imputed and true p-values (y-axis: −log10(P_method_/P_nomiss_)). Panel A shows results from the dosage-based method, while Panel B illustrates results obtained using the entropy-weighted method. Panels C and D show the distribution of p-value discordance stratified by imputation quality (R^2^) for the dosage and entropy-weighted methods, respectively. Data were generated from simulations with a SNP density of approximately 2,500 SNPs/Mb (corresponding to 90% masking relative to the full 1000 Genomes reference panel). Positive discordance values indicate inflation of imputed p-values compared to the “true” (non-masked) dataset.

Figure 4 presents the results from input genotype data with a SNP density of approximately 2,500 SNPs/Mb, corresponding to a 90% masking rate relative to the full 1000 Genomes reference panel. Panels A and C display results obtained using the conventional dosage-based association method, while Panels B and D illustrate results using our proposed entropy-weighted method. We assessed the discordance between association p-values derived from the complete dataset (P_nomiss_) and those obtained using either the dosage (P_dosage_) or entropy-weighted (P_weight_) methods. The discordance was quantified as −log10(P_method_/P_nomiss_), where positive values reflect inflation in imputed p-values. In Panel A, the discordance between the association p-values derived from imputed dosages and those from the complete (non-masked) dataset increases markedly as p-values approach zero. This indicates that SNPs with the strongest signals are also most susceptible to distortion due to imputation uncertainty. By contrast, Panel B shows that applying the entropy-weighted method significantly mitigates this effect: although some discordance remains for low p-values, both the magnitude and frequency of discordant SNPs are substantially reduced. Notably, the peaks in the distribution of discordance degrees decrease by roughly half, suggesting improved stability and reliability in signal ranking. Panels C and D further stratify discordance by imputation quality (as measured by allelic R^2^). In the dosage-based analysis (Panel C), discordant SNPs are observed across a wide range of R^2^ values, including those with poor imputation quality. In contrast, Panel D reveals that the entropy-weighted method naturally downweights or excludes low-quality SNPs, as evidenced by the sharp drop in discordant SNPs with decreasing R^2^. This pattern highlights a key advantage of our method: it adaptively reduces the influence of uncertain imputation calls without the need for hard filtering, thereby preserving statistical power while controlling false discoveries.

**Figure 5:**
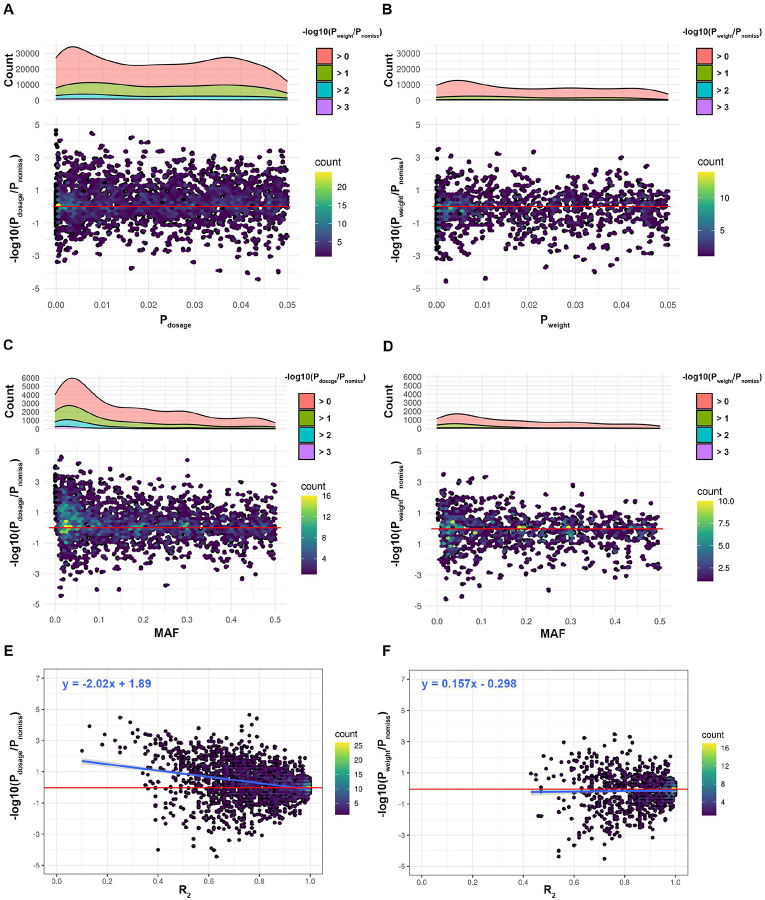
Discordance of p values in an extreme sparse dataset The hexagonal bins represent the discordance between anticipated p-values and dosage p-values (P_dosage_) in panels A, C, and E, as well as weighted p-values (P_weight_) in panels B, D, and F. Notably, the p values of in this figure was adjusted using the false discovery rate (FDR) method, and the figure exclusively exhibits data points where imputed p-values (P_dosage_ in A, C, and E, P_weight_ in B, D, and F) are < 0.05. The color variation within each hexagonal bin reflects the range of discordance values encapsulated by that bin. In a top-to-bottom progression, the panels showcase the interplay between the discordance and either P_dosage_ (A) or P_weight_ (B), and the minor allele frequency (MAF) (C, D), and the imputation quality metric R2 (E, F). Superimposed density plots on the upper part of panels A-D illustrate the distribution of positive discordant values (associated with the inflation of imputed p-values) along the x-axis, categorized by distinct levels of discordance. Red horizontal lines underscore instances of discordance at a value of 0, and blues lines in (E and F) are the best linear fit, providing an illustrative depiction of the alignment between data points. Note that results shown in this figure are from the input data with an approximate SNPs density of 50 SNPs/Mb.

Our entropy-weighted method continued to outperform the conventional dosage-based approach even under conditions of extreme genotype sparsity. Figure 5 illustrates results from simulations with a SNP density of only 50 SNPs/Mb (equivalent to a 99% masking rate relative to the full 1000 Genomes panel). The discordance of p-values is derived using the same method as in Figure 4. In Panels A and C of Figure 5, the dosage method produced widespread p-value inflation, especially among SNPs with small P_dosage_ values, where inflated values exceeded 3 log-fold changes. This inflation was particularly pronounced in low-p-value regions, indicating systematic overestimation of significance under sparse input conditions. In contrast, Panels B and D reveal that the entropy-weighted method substantially reduced both the frequency and magnitude of p-value inflation. The peaks of the frequency distributions of discordance values were visibly suppressed, and the magnitude of discordance remained more tightly constrained, even in the presence of low minor allele frequency (MAF) variants. This demonstrates that our method not only improves robustness but also maintains calibration across the full MAF spectrum.

Panels E and F of Figure 5 explore the relationship between p-value discordance and imputation quality as measured by allelic R^2^. While it is standard practice to apply an R^2^ threshold to remove poorly imputed SNPs, Panel E illustrates that p-value inflation in the dosage method is strongly correlated with low R^2^, necessitating aggressive filtering. By contrast, Panel F shows that the entropy-weighted method yields p-values that are tightly centered around zero discordance across a broad range of R^2^ values. Notably, the majority of SNPs retained by the entropy-based method had R^2^>0.8, while very few SNPs with poor imputation quality (R^2^<0.4) were included. This further suggests that our approach inherently downweights or excludes uncertain genotypes, effectively controlling inflation without requiring explicit post-imputation filtering based on quality metrics.

**Figure 6.**
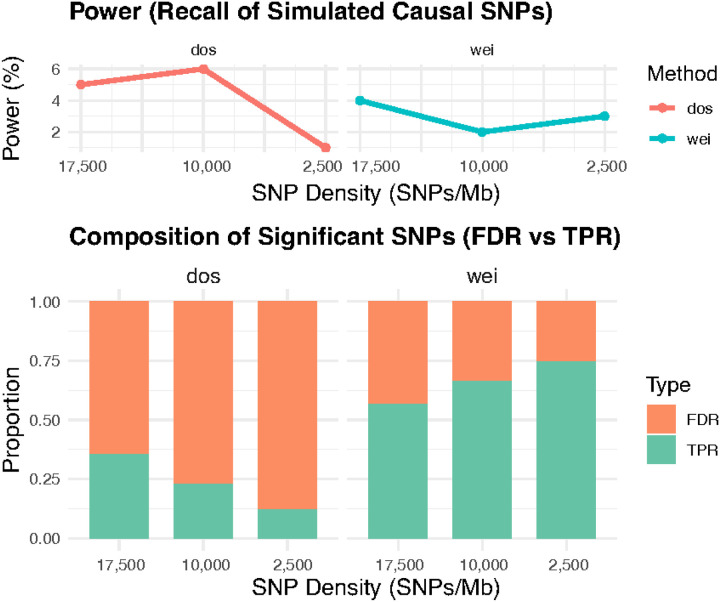
Comparison of Power and False Discovery Rate Across Genotype Masking Rates This figure evaluates the performance of the dosage-based (dos) and entropy-weighted (wei) association methods under varying levels of genotype masking. In the top row, the line plots depict the power (recall rate) for each method to detect 100 simulated causal variants as a function of the SNP density. Power is expressed as the percentage of true causal variants successfully identified at an FDR-adjusted significance threshold of p < 0.05. The bottom row presents the composition of significant SNPs identified by each method at different SNP density. Stacked bar plots show the proportion of significant SNPs corresponding to true positives (TPR, causal variants) and false positives (FDR, non-causal variants).

We further applied the Benjamini–Hochberg false discovery rate (FDR) correction^26^ to P_nomiss_, P_dosage_, and P_weight_ to identify significant signals for each method. To evaluate FDR and true positive rate (TPR), we simulated 100 causal variants based on 1000G genotype data and compared the performance of the dosage-based and entropy-weighted approaches in the simulation setting. In Figure 6, we first assess the power of each method to recover the 100 simulated causal variants. As shown in the top two panels, the power for both methods was generally low, with recall rates below 10%. This limited power likely reflects multiple factors, such as relatively small effect sizes, low MAF of SNPs, or insufficient sample size. Overall, the entropy-weighted method exhibited lower power compared to the dosage method. For instance, at an SNP density of approximately 5,000 SNPs/Mb, the dosage method’s power was three times higher than that of the weighted method. However, because overall power was very limited and only a small number of causal variants were successfully recalled, these power differences may fluctuate by chance, making it difficult to confidently determine a trend across varying SNP densities.

In the bottom panel of Figure 6, we further evaluated FDR and TPR by counting the number of causal variants identified at an adjusted p-value threshold of p < 0.05. We observed that the entropy-weighted method consistently exhibited a lower FDR than the dosage method across all scenarios. Importantly, as the genotype data became sparser, the FDR patterns diverged between methods: while the FDR of the dosage method increased with decreasing SNP density, the FDR of the entropy-weighted method decreased. For example, when SNP density declined from ~17,400 SNPs/Mb to ~2,500 SNPs/Mb, the FDR of the dosage method increased from 0.64 to 0.88, whereas the FDR of the weighted method decreased from 0.43 to 0.25. This may be because, under extreme sparsity, a larger fraction of imputed SNPs possess lower imputation quality, and the entropy-based method can effectively downweight these uncertain genotypes. As demonstrated in Figures 4 and 5, the entropy-weighted approach systematically suppresses poorly imputed SNPs, thereby substantially reducing the false discovery rate.

**Figure 7.**
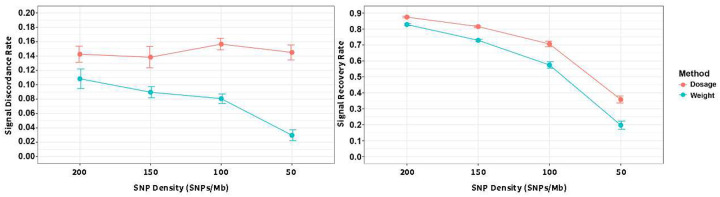
Signal Discordance and Recovery Rates Across SNP Density Levels Under Extreme Sparsity The p-values obtained from analyses of complete genotype data (P_nomiss_), dosage-based imputed data (P_dosage_), and entropy-weighted imputed data (P_weight_) were adjusted using the Benjamini–Hochberg procedure to control for multiple testing. SNPs identified as significant (adjusted P_nomiss_ <0.05) in the complete genotype dataset formed the reference signal set. Panel A shows the signal discordance rate, defined as the proportion of significant SNPs (adjusted P<0.05) identified in imputed datasets (dosage and entropy methods) that were not part of the reference signal set. Panel B illustrates the signal recovery rate, defined as the proportion of the reference signal set successfully recovered in each imputed dataset. Results are summarized across simulations spanning multiple SNP densities (200 to 50 SNPs/Mb), corresponding to different genotype masking scenarios.

Our method continued to perform robustly even in extremely sparse datasets. In Figure 7, rather than referring to “true positives” in the strict statistical sense, we used SNPs with adjusted P_nomiss_ < 0.05 as a reference signal set, and assessed how well this set was recovered by the dosage-based and entropy-weighted methods under varying levels of SNP density. This approach enables an evaluation of each method’s ability to preserve significant associations in the presence of imputation uncertainty. As shown in Figure 7A, as SNP density decreased from approximately 200 SNPs/Mb to 50 SNPs/Mb, the discordant recovery rate — analogous to an empirical false discovery rate — fluctuated between 14–16% for the dosage method. In contrast, the entropy-weighted method showed a sharp reduction in discordance, with non-recovery rates decreasing from 11% to just 3% under extreme sparsity. These results illustrate the entropy-weighted method’s superior ability to maintain signal consistency and mitigate inflation of false associations under high genotype uncertainty. Figure 7B further explores the proportion of reference signals that were successfully recovered. While signal recovery declined for both methods with increasing missingness, the entropy-weighted method showed a more substantial drop: recovery decreased from 83% at ~200 SNPs/Mb to 20% at ~50 SNPs/Mb, compared to the dosage method, which declined from 86% to 36%. This sharper decline reflects the more stringent downweighting of low-confidence genotypes by the entropy method, as previously observed in Figures 4 and 5. Despite the trade-off of reduced sensitivity, this strategy yields a substantial gain in signal specificity and reduction of p-value inflation, highlighting the entropy-weighted method’s strength in controlling false discoveries when genotype uncertainty is high.

## Discussion

Genotype imputation is increasingly employed in genetic studies to enhance analytical power. Recent advancements such as low-coverage whole-genome sequencing (lcWGS) have provided a cost-effective means to sequence large cohorts, and it inherently produces sparse and incomplete genotype calls that depend heavily on accurate imputation to achieve sufficient variant density for robust genetic analyses. Concurrently, the growth of dense reference panels, notably the 1000 Genomes Phase III panel^21^ (with approximately 49 million variants from 2,504 individuals) and the Haplotype Reference Consortium (HRC)^27^ panel (nearly 40 million variants from 2,470 individuals), has significantly enhanced imputation accuracy. Despite these improvements and strategies such as employing extensive reference panels and excluding genotypes with poor imputation quality, considerable challenges remain, particularly in controlling false discoveries in genome-wide association studies (GWAS) conducted on sparse datasets.^19^

For instance, when imputing genotyping arrays such as the UK Biobank, which initially comprises roughly 850,000 variants—further reduced after quality control—over 90% of genotypes must typically be imputed. This large proportion of missing data substantially reduces imputation quality and biases downstream association analyses. The conventional approach in GWAS involves summarizing probabilistic genotypes into allele dosage values, collapsing posterior genotype probabilities into a single dimension. However, as illustrated in our analysis, this dosage-based approach disregards crucial differences in genotype uncertainty: for example, a dosage value around 1.0 could correspond to numerous distinct probability distributions of genotypes (AA, Aa, aa), each carrying unique uncertainty levels. Such oversimplification inevitably leads to biased estimates and inflates false-positive findings, especially in sparse datasets where genotype uncertainty is inherently greater.

Our entropy-weighted approach assigns observation-specific weights that reflect the confidence in genotype calls, effectively differentiating between genotype sets that share similar dosage values but differ significantly in uncertainty. Through simulation studies, we demonstrated that the entropy-weighted method markedly reduces false discoveries compared to the traditional dosage method. This improvement was consistent across diverse scenarios, including variants with varying minor allele frequencies (MAF) and differing imputation qualities (R^2^).

Importantly, our simulations incorporated both dense genotype data from the 1000 Genomes Project and extremely sparse genotype data derived from the UK Biobank, representing realistic extremes of current genotyping practices. Even in simulations representing extreme sparsity—down to approximately 50 SNPs/Mb—our entropy-weighted method consistently outperformed the dosage-based approach, maintaining robust control over false-positive rates. This level of sparsity reflects the lower bound of variant density found in early GWAS designs, such as the Affymetrix GeneChip 100K array^28^, which was used in some of the earliest large-scale genetic studies. Such sparse datasets are typically challenging to impute with high confidence and are prone to inflated false discoveries when using conventional methods. By explicitly modeling genotype uncertainty, our entropy-based framework provides a principled way to rescue and repurpose these legacy or budget-constrained datasets, enabling them to yield reliable association signals despite their limited resolution. Additionally, our approach inherently excludes variants with poor imputation quality, effectively removing the need for stringent, manually defined post-imputation quality filters. Although applying the entropy-weighted method did result in some reduction of true positive discovery rates, the magnitude of reduction in false positives substantially outweighed this loss, yielding a significant net gain in reliability and interpretability of GWAS results.

While this study primarily evaluated binary traits using the UK Biobank and 1000 Genomes datasets, our entropy-based weighting framework is readily adaptable to quantitative traits as well. For quantitative outcomes, our weighting strategy can be seamlessly incorporated into a modified log-likelihood framework, for instance:

$$l=\frac{1}{2}\sum_{i=1}^{n}w_{i}{\left(y_{i}-\mu_{i}\right)}^{2}$$

This flexibility suggests broader applicability of our approach across various trait types and analytical frameworks within genetic epidemiology.

## Conclusions

In summary, our study introduces and rigorously validates an innovative entropy-based weighting strategy that addresses the common bias in genotype imputation based GWAS. By explicitly accounting for the confidence level of imputed genotype calls, our approach significantly enhances the reliability and accuracy of association analyses in GWAS, even in highly sparse datasets characterized by substantial genotype uncertainty. This methodology not only improves imputation-based GWAS but also provides a framework for future genetic studies to better leverage the rich yet inherently uncertain data generated by modern genotyping and sequencing technologies.

## Supplementary Material

Supplementary Files

This is a list of supplementary files associated with this preprint. Click to download.

- sup1.png

## Data Availability

This study uses existing data from publicly available resources. The 1000 Genomes Project data are publicly accessible at the International Genome Sample Resource (IGSR) (https://www.internationalgenome.org/data). Access to UK Biobank data is available upon application and approval from the UK Biobank (https://www.ukbiobank.ac.uk/enable-your-research/apply-for-access). No new datasets were generated in this study.
